# Adjuvantation of Pulmonary-Administered Influenza Vaccine with GPI-0100 Primarily Stimulates Antibody Production and Memory B Cell Proliferation

**DOI:** 10.3390/vaccines5030019

**Published:** 2017-07-27

**Authors:** Harshad P. Patil, José Herrera Rodriguez, Jacqueline de Vries-Idema, Tjarko Meijerhof, Henderik W. Frijlink, Wouter L. J. Hinrichs, Anke Huckriede

**Affiliations:** 1 Department of Medical Microbiology, University of Groningen, University Medical Center Groningen, Antonius Deusinglaan 1, 9713 AV Groningen, The Netherlands; mailharshadpatil@gmail.com (H.P.P.); j.herrera.rodriguez@umcg.nl (J.H.R.); j.j.de.vries-idema@umcg.nl (J.d.V.-I.); t.meijerhof@umcg.nl (T.M.); 2 Department of Communicable Diseases, Interactive Research School for Health Affairs, Bharati Vidyapeeth University, Pune-Satara Road, Katraj-Dhankawadi, Pune 411043, Maharashtra, India; 3 Department of Pharmaceutical Technology and Biopharmacy, University of Groningen, Antonius Deusinglaan 1, 9713 AV Groningen, The Netherlands; h.w.frijlink@rug.nl (H.W.F.); w.l.j.hinrichs@rug.nl (W.L.J.H.)

**Keywords:** influenza, pulmonary immunization, adjuvant, immune mechanisms

## Abstract

Adjuvants are key components in vaccines, they help in reducing the required antigen dose but also modulate the phenotype of the induced immune response. We previously showed that GPI-0100, a saponin-derived adjuvant, enhances antigen-specific mucosal and systemic antibody responses to influenza subunit and whole inactivated influenza virus (WIV) vaccine administered via the pulmonary route. However, the impact of the GPI-0100 dose on immune stimulation and the immune mechanisms stimulated by GPI-0100 along with antigen are poorly understood. Therefore, in this study we immunized C57BL/6 mice via the pulmonary route with vaccine consisting of WIV combined with increasing amounts of GPI-0100, formulated as a dry powder. Adjuvantation of WIV enhanced influenza-specific mucosal and systemic immune responses, with intermediate doses of 5 and 7.5 μg GPI-0100 being most effective. The predominant antibody subtype induced by GPI-0100-adjuvanted vaccine was IgG1. Compared to non-adjuvanted vaccine, GPI-0100-adjuvanted WIV vaccine gave rise to higher numbers of antigen-specific IgA- but not IgG-producing B cells in the lungs along with better mucosal and systemic memory B cell responses. The GPI-0100 dose was negatively correlated with the number of influenza-specific IFNγ- and IL17-producing T cells and positively correlated with the number of IL4-producing T cells observed after immunization and challenge. Overall, our results show that adjuvantation of pulmonary-delivered WIV with GPI-0100 mostly affects B cell responses and effectively induces B cell memory.

## 1. Introduction

Adjuvants are important components of vaccines as they have the ability to enhance and to modify the immune responses induced against an antigen. Many studies are ongoing to find effective mucosal adjuvants and to elucidate the mechanisms by which they can help to reduce the antigen content in a vaccine without compromising the immune response induced against the antigen itself. GPI-0100 is one of such adjuvants; it is under preclinical investigation for use in parenteral and pulmonary vaccines [1,2], and has been used in clinical tumor immunotherapy trials [3,4].

GPI-0100 is a semisynthetic triterpenoid saponin prepared by first deacylating a mixture of *Quillaja saponaria* saponins and then coupling dodecylamine with the carboxyl group of the glucuronic acid residue of the deacylated saponins through an amide bond [5]. GPI-0100 is a highly purified analogue of QS-7, which also has immune-modulating properties [5,6]. GPI-0100 is more stable than other *Quillaja* saponins and has a better safety profile [4]. The receptor for GPI-0100 is not known; however, its adjuvant activity is believed to be mediated by the aldehyde group of the molecule and might be related to its capacity to form pores in the lipid bilayer of cells. GPI-0100 has been shown to stimulate Th1 immunity, cytotoxic T lymphocytes (CTL) responses, and antibody production against co-delivered antigens [7]. Use of GPI-0100 as adjuvant for parenteral subunit or virosomal influenza vaccines allowed induction of robust and protective immune responses in mice even at very low antigen doses (8 ng) [1,2].

An attractive alternative to parenteral vaccination is pulmonary vaccine delivery. Pulmonary vaccination is easy to perform, patient-friendly and capable of inducing immune responses at the portal of entry of many pathogens [8]. Pulmonary vaccine delivery targets the lungs, which form a highly vascularized organ with a large surface area that is under constant immune surveillance. Several small- to large-scale human clinical trials demonstrate that pulmonary immunization in humans is safe and feasible [9,10]. Recently, inhaled live attenuated measles vaccine formulated as aerosol or dry powder was demonstrated to be safe and effective in a Phase I clinical trial [11].

The suitability of saponin-derived adjuvants for pulmonary immunization was first studied in sheep by Wee and co-workers [12]. These authors showed that ISCOMATRIX, an adjuvant composed of purified fractions of *Quillaia saponaria* extract (ISCOPREP saponin) along with cholesterol and phospholipid, induced markedly increased lung and serum antibody titers to whole inactivated virus (WIV) influenza vaccine that was delivered to the lower caudal lobe of the sheep lung. In addition, ISCOMATRIX-adjuvanted pulmonary vaccine also induced long-term antibody and memory responses [13]. We have earlier shown that immune responses to pulmonary-delivered influenza subunit or WIV vaccine in mice could be significantly enhanced by inclusion of GPI-0100 as adjuvant in both liquid and dry powder vaccine formulations [14,15]. Recently, we investigated in a head-to-head comparison in mice four different adjuvants for a pulmonary-delivered WIV influenza vaccine. Compared to the TLR ligands Pam3CSK4, MPLA and CpG, GPI-0100 was more potent in inducing mucosal and serum antibodies. Moreover, mice immunized with WIV-GPI-0100 showed reduced lung virus titers after challenge with heterologous influenza strain [15].

In this study, we evaluated in more detail the immune mechanisms induced by pulmonary-delivered GPI-0100-adjuvanted influenza vaccine. To this end, we immunized mice twice with WIV vaccines containing different doses of GPI-0100 and subsequently challenged them with live virus. The vaccines were formulated as dry powders and were administered to the trachea of intubated mice. Systemic and mucosal antibody responses as well as numbers of germinal center and memory B cells, and cytokine-producing T cells were determined. Our results reveal that GPI-0100 in combination with WIV mainly affects B cells and that an intermediate dose of the adjuvant is most effective.

## 2. Materials and Methods

### 2.1. Virus and Vaccine Preparation

Influenza strain A/Puerto Rico/8/34 H1N1 (A/PR/8) was cultured in embryonated eggs and WIV was produced by treating the sucrose gradient purified virus with 0.1% β-propiolactone as described previously [16,17]. GPI-0100 was purchased from Hawaii Biotech (Honolulu, HI, USA). GPI-0100-adjuvanted vaccine solutions were prepared by adding different amounts of GPI-0100 to WIV vaccine solution (WIV:GPI-0100, *w*/*w*, 3:1, 3:2, 3:3 and 3:6). Vaccine solutions were later mixed with inulin solution to obtain a final concentration of 5% *w*/*v* inulin resulting in a protein:inulin ratio of 3:200 *w*/*w*. This mixture was spray freeze dried as described previously [18].

### 2.2. Immunization, Challenge and Sample Collection

Animal experiments were approved by The Institutional Animal Care and Use Committee of the University of Groningen (IACUC-RuG), The Netherlands (Ethical protocol number DEC 5879I). In vivo experiments were performed in 6–8-week-old female C57BL/6 mice (Harlan, Zeist, The Netherlands).

Mice (n = 6/experimental group) were anaesthetized using isoflurane/O_2_ and vaccinated twice via the pulmonary route, with an interval of 3 weeks as described previously [18]. Briefly, mice were intubated using a modified Autoguard catheter (Becton Dickinson, Breda, The Netherlands) and approximately 500 μg of powder vaccine containing 2.5 μg HA of A/PR/8 was delivered to the lungs by applying a single puff of 200 μL using a dry powder insufflator (DPI, Penn-Century Inc., Wyndmoor, PA, USA). Mice sublethally infected with A/PR/8 served as positive control. Sublethal infection was done by gentle administration of 1000 TCID_50_ A/PR/8 in 2.5 μL phosphate buffered saline (PBS) to the nostrils. Mice infected twice in this way developed robust antibody titers but no disease symptoms. Untreated mice served as negative control.

Thirty days after the booster vaccination or the second sublethal infection, the effect of the vaccines/previous infection on disease parameters was determined by total respiratory tract challenge of the mice. To this end, a dose of 100 TCID_50_ A/PR/8 virus (10 LD_50_) was administered intranasally in a total volume of 40 μL PBS as described previously [17]. Five days post challenge, mice were sacrificed under isoflurane/O_2_ anesthesia. Blood, nose washes, bronchoalveolar lavages (BAL), lungs and spleen were collected. Nose wash and BAL were collected using 1 mL PBS containing complete protease inhibitor cocktail (Roche, Almere, The Netherlands). Lungs were perfused through the heart using 20 mL PBS containing heparin. The right lobes of the lungs were collected in PBS with protease inhibitor for virus quantification and stored on ice. The remaining parts of the lungs were collected in complete Iscove’s Modified Dulbecco’s Medium (IMDM) with 1 mg/mL collagenase D (Roche, Almere, The Netherlands) and processed as described previously to assess lymphocytes [19]. Spleens were collected in complete IMDM and were processed to single-cell suspension using GentleMACS C tubes and a GentleMACS dissociator (Mitenyi Biotec B, Leiden, The Netherlands) and stored in ice. Later, red blood cells were lysed using ACK buffer (0.83% NH_4_Cl, 1 mM KHCO_3_, 0.1 mM EDTA, pH 7.2). ELISpot and flow cytometry were performed using splenocytes from individual mice or lung lymphocytes pooled per experimental group.

### 2.3. ELISA

Two hundred microliter of nose washes and bronchoalveolar lavages (BAL) were used for analysis of mucosal antibody responses. Serum samples were diluted 1:100 and 200 μL was used for evaluation of humoral immune responses. Influenza-specific IgG, IgG1, IgG2c and IgA antibody titers were determined by ELISA as previously described [18]. IgG titers were calculated as the reciprocal of the dilution resulting in an optical density (OD) of 0.2. Since for IgA in nose wash an OD of 0.2 was not reached in all cases, a titer could not be calculated; antibody amounts are depicted as OD492 values instead.

### 2.4. Virus Titration

Collected lungs were homogenized in PBS (pH 7.4) followed by centrifugation at 1200 rpm for 10 min at 4 °C. Lung homogenate supernatants were collected, snap-frozen in liquid nitrogen and stored at −80 °C until use. Lung virus titers were determined by infecting MDCK cells grown in 96-well plates as described previously [16]. Virus titers are presented as log_10_ titer per gram lung tissue.

### 2.5. ELISpot

B cell ELISpot was performed as previously described with some modifications [20]; 5 × 10^5^ lymphocytes from lungs or splenocytes were added to MultiScreen_HTS_-HA filter plates (Millipore, Billerica, MA, USA) uncoated or coated with 10 μg/mL A/PR/8 subunit vaccine. Plates were incubated overnight at 37 °C with 5% CO_2_ in IMDM complete medium. Subsequently, alkaline phosphatase labeled anti-mouse IgA antibody (Sigma-Aldrich Chemie B.V., Zwijndrecht, The Netherlands) or horse radish peroxidase labeled anti-mouse IgG antibody (Southern Bio-tech, Birmingham, AL, USA) was added to the wells. The numbers of IgA- and IgG-secreting cells (ASC) were quantified using 5-bromo-4-chloro-3′-indolyphosphate p-toluidine/nitro-blue tetrazolium chloride (BCIP/NBT) and 3-amino-9-ethylcarbazole (AEC) substrate (Roche, Almere, The Netherlands), respectively.

Influenza-specific IFNγ-, IL4- and IL17-producing T cells were enumerated by ELISpot using a murine IFNγ ELISpot kit (Gen-Probe Diaclone SAS, Besancon Cedex, France), an in-house IL4 ELISpot protocol, or an IL17 ELISpot kit (eBioscience, Vienna, Austria), respectively. Briefly, 5 × 10^5^ lymphocytes from lungs or splenocytes were added to MultiScreen_HTS_-HA filter plates (Millipore, Billerica, Massachusetts) coated with anti-IFNγ, anti-IL4 (BD Biosciences, Breda, The Netherlands) or anti-IL17 antibodies. Plates were incubated overnight at 37 °C with 5% CO_2_ in IMDM complete medium without or with 10 μg/mL WIV obtained from A/PR/8. For IFNγ and IL17 ELISpot, staining was done as per the manufacturer’s instructions. IL4-producing cells were detected using alkaline phosphatase-labelled anti-mouse IL4 antibodies (eBioscience, Vienna, Austria). Plates were washed with PBS and spots were developed using BCIP/NBT substrate (Roche, Almere, The Netherlands). The reaction was stopped by washing plates with tap water.

### 2.6. Flow Cytometry

To U-bottom 96-well plates (Corning Incorporated, New York, NY, USA) 1 × 10^6^ splenocytes or lung lymphocytes were added per well. After extensive washing with PBS, containing 0.1% bovine serum albumin, cells were centrifuged at 1200 rpm for 5 min at 4 °C and blocked with Fc Block (BioLegend, San Diego, CA, USA). Pelleted cells were resuspended and for B cell analysis stained with anti-IgM PeCy7, anti-GL7 eFluoro 660, anti-CD38 eFluro 450 (all antibodies from eBioscience, Vienna, Austria), anti-IgD PeCy7, anti-CD138 PerCpCy5.5 and anti-B220 BV605 (BioLegend, San Diego, CA, USA) diluted in FACS buffer at 4 °C for 60 min. Cells were then washed and analyzed using a BD FACS Verse^TM^ flow cytometer (BD Biosciences, Breda, The Netherlands). Obtained data was analyzed using Kaluza flow cytometry analysis software version 1.2 (Beckman Coulter, Woerden, The Netherlands).

### 2.7. Statistical Analysis

Since the data do not follow Gaussian distribution, the non-parametric Mann–Whitney U test was used. One research question was to study whether addition of GPI-0100 to WIV results in an increase of the humoral response. With the null hypothesis being that there is no statistically significant increase in humoral responses by the GPI-0100 dose in question, we performed a one-tailed Mann–Whitney U test for comparison of mice immunized with different amounts of GPI-0100 versus the non-adjuvanted vaccine. Two-tailed Mann–Whitney test was used to analyze cytokine-producing cells with null hypothesis being there is no statically significant increase or decrease in various cytokine-producing cells by GPI-0100. *p* values < 0.05 were considered to represent statistically significant differences; * and ** signify *p* < 0.05 and *p* < 0.01, respectively.

## 3. Results

### 3.1. Systemic Antibody Responses

**Figure A1:**
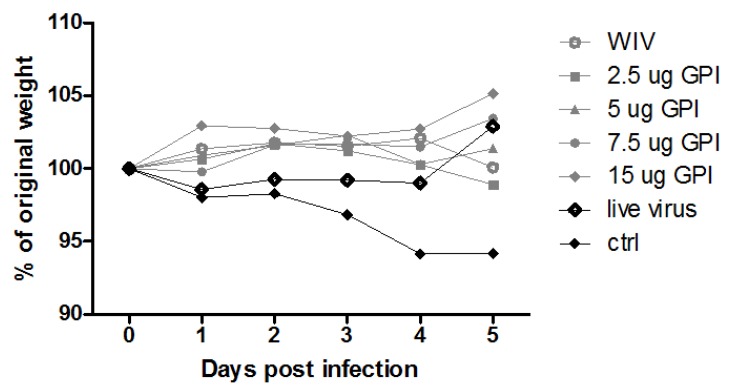
Weight development after challenge. Mice (n = 6 per group) were immunized via the pulmonary route on day 0 and 21 with powder vaccines containing A/PR/8 WIV (2.5 μg HA) alone or with the indicated amounts of GPI-0100. As a positive control, mice were sub-lethally infected by nose-restricted administration of 1000 TCID_50_ A/PR/8 on day 0 and 21. On day 51, mice were challenged with 100 TCID_50_ of A/PR/8 administered to the total respiratory tract and weight was monitored daily until sacrifice on day 5. Open circles: WIV, filled grey circles: WIV + GPI-0100 (increasing adjuvant dose = darker shades), black circle: previously infected mice, open squares: naïve control.

Previous studies on pulmonary delivery of GPI-0100 along with influenza subunit or WIV vaccine demonstrated that GPI-0100 is a potent adjuvant for induction of humoral responses [14]. To find out more about the effect of GPI-0100 dose and how it affects the type of immune responses, in this study, mice were immunized twice with WIV alone or WIV admixed with different amounts of GPI-0100. Mice infected intranasally with a sub-lethal amount of virus served as a positive control. On day 28 after the last immunization, the mice were challenged with A/PR/8 virus. On day 5 after challenge, the day of sacrifice, all naïve control mice had substantial amounts of virus in the lung (average log_10_ virus titer/g lung tissue 5.5 ± 0.22 SEM) and all but one showed weight loss (Appendix Figure A1). In contrast, all immunized animals, except one mouse immunized with non-adjuvanted WIV, were completely protected from virus growth in the lungs. In none of the immunized groups did we observe statistically significant weight loss over the infection period, although in the WIV, 2.5 μg GPI, and 5 μg GPI group 3, 1 and 2 out of 6 mice, respectively, experienced some weight loss. Thus, irrespective of the use of adjuvants pulmonary immunizations with WIV elicited immune responses which protected the mice from virus growth in the lungs and from clinical symptoms.

**Figure 1:**
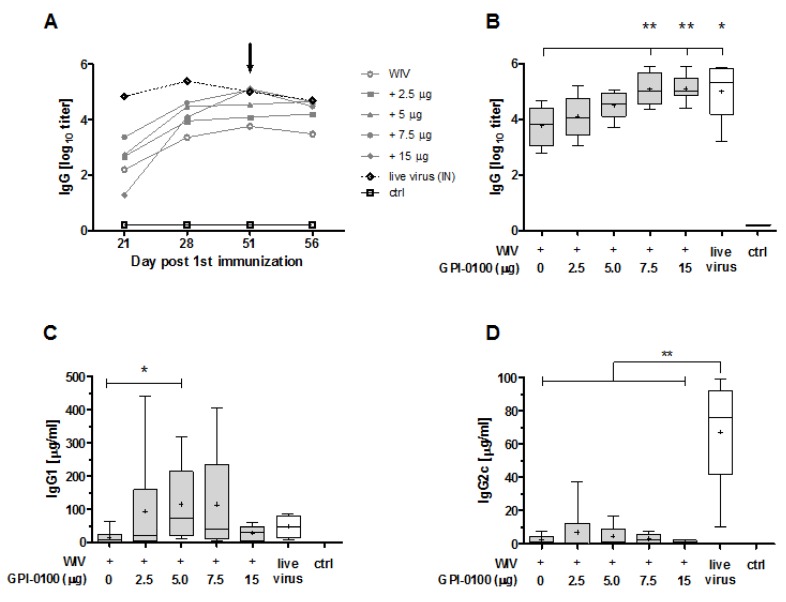
Effect of GPI-0100 on systemic antibody responses. Mice (n = 6 per group) were immunized via the pulmonary route on day 0 and 21 with powder vaccines containing A/PR/8 whole inactivated virus (WIV) (2.5 μg HA) alone or with the indicated amounts of GPI-0100. As a positive control, mice were sub-lethally infected by nose-restricted administration of 1000 TCID_50_ A/PR/8 on day 0 and 21. Control mice (ctrl) were non-immunized. On day 51, all mice were challenged with 100 TCID_50_ of A/PR/8 administered to the total respiratory tract. (**A**) A/PR/8-specific IgG responses evaluated in serum collected on days 21, 28, 51 and 56. Mean group titers calculated from individual titers are depicted. Arrow indicates day of challenge. (**B**) IgG, (**C**) IgG1 and (**D**) IgG2c after two immunizations. IgG antibody levels are expressed as log_10_ titers. IgG1 and IgG2c levels are shown as μg/mL. Results in B, C and D are shown as box and whisker plots depicting the median, mean, maximum and minimum of the group. Levels of significance are presented as * *p* < 0.05, ** *p* < 0.01.

**Figure A2:**
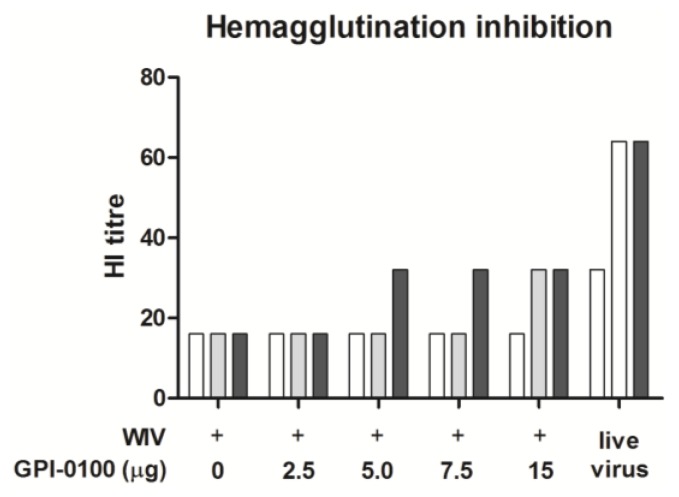
Hemagglutination inhibition titers. Mice (n = 6 per group) were immunized via the pulmonary route on day 0 and 21 with powder vaccines containing A/PR/8 WIV (2.5 μg HA) alone or with the indicated amounts of GPI-100. As a positive control, mice were sub-lethally infected by nose-restricted administration of 1000 TCID_50_ A/PR/8 on day 0 and 51. A/PR/8-specific hemagglutination inhibition titers were evaluated in serum extracted from blood collected on day 21 (white bars), 51 (grey bars), and 5 days after virus challenge, i.e., on day 56 (black bars). Results for pooled sera per experimental group are shown.

After a single administration, none of the vaccines was able to induce IgG antibodies to the levels found in mice given a sub-lethal influenza virus infection which served as a positive control (Figure 1A, day 21). However, after the booster dose, which was given 21 days after the first dose, there was an increase in IgG titers by 1–3 log in all immunized mice. By day 51, IgG levels in mice immunized with non-adjuvanted WIV were still significantly lower than in convalescent mice (*p* = 0.0411). GPI-0100 increased vaccine-induced IgG titers in a dose-dependent manner; by day 28 (7.5 μg GPI-0100) or day 51 (all adjuvanted vaccines), IgG levels in mice immunized with adjuvanted vaccines no longer differed significantly from those in convalescent mice. Moreover, IgG levels elicited by vaccines adjuvanted with 7.5 or 15 μg GPI-0100) were significantly higher than those elicited by non-adjuvanted vaccine (Figure 1B). No increase in IgG antibody titers was observed 5 days after virus challenge, i.e., on day 56, neither in naïve nor in vaccinated animals. In line with previous observations with A/PR/8 vaccines [14], HI titers were generally low (<40) even after two immunizations (Appendix Figure A2). HI titers increased slightly after challenge in the 5, 7.5 and 15 μg GPI-0100 groups but not in the 2.5 μg GPI-0100 group and the group immunized with non-adjuvanted vaccine.

Quantification of IgG subclasses (Figure 1C,D) demonstrated that mice immunized with GPI-0100-adjuvanted formulations developed higher levels of influenza-specific IgG1 antibody than mice immunized with non-adjuvanted vaccine. Enhancing effects of GPI-0100 were highest for the 5 and 7.5 μg GPI-0100 groups; unexpectedly, mice immunized with 15 μg GPI-0100 had rather low levels of IgG1. However, the variation within the experimental groups was high and the difference in IgG1 levels as compared to the group immunized with non-adjuvanted vaccine was significant only for the 5 μg GPI-0100 group. Analysis of IgG2c levels revealed that GPI-0100 had hardly any effect on induction of this antibody subclass irrespective of the GPI-0100 dose used; most of the mice given GPI-0100 adjuvanted WIV had IgG2c levels similar to those induced by non-adjuvanted WIV. In contrast, sub-lethally infected mice developed robust IgG2c responses which exceeded IgG1 levels in these animals. No influenza-specific IgG1 or IgG2c were found in the non-immunized control mice.

### 3.2. Mucosal Antibody Responses

**Figure 2:**
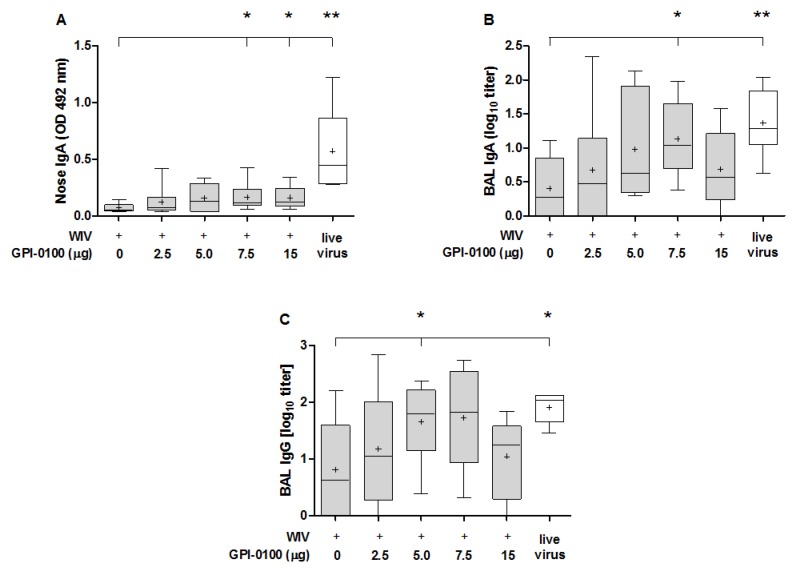
Effect of GPI-0100 on antibody responses in respiratory tract. Mice (n = 6) were vaccinated and challenged as described in the legend to Figure 1. Nose washes and bronchoalveolar lavages (BAL) were collected 5 days after challenge with A/PR/8, i.e., on day 56 and were analyzed for (**A**) nose IgA; (**B**) BAL IgA; and (**C**) BAL IgG. IgA responses in the nose wash are presented as absorbance at OD492 while BAL IgA and IgG antibody levels are expressed as log_10_ titers. Live virus = sublethally infected, challenged mice. Ctrl = non-immunized but challenged mice. Results are shown as box and whisker plots depicting the median, mean, minimum and maximum of the experimental group. Levels of significance are presented as * *p* < 0.05, ** *p* < 0.01.

The ability of GPI-0100 to boost the mucosal antibody response was evaluated in nose wash and BAL of immunized and challenged mice (Figure 2). Quantification of antibody in the nose washes (Figure 2A) showed that GPI-0100 positively affected nasal IgA responses. However, the enhancing effect was limited and reached significance only in the 7.5 and 15 μg GPI-0100 groups. Irrespective of the use of adjuvant, the vaccine-induced nose IgA responses were much lower than the responses induced by infection. A similar trend as for nose IgA titers was observed for IgA and IgG antibodies in the BAL (Figure 2B,C). Mean titers increased up to a dose of 7.5 μg GPI-0100; differences as compared to titers in the WIV-alone group being significant for the 7.5 μg group (BAL IgA) and 5 μg group (BAL IgG). The amount of IgA present in the lung was much higher than in the nose. Moreover, BAL IgA and IgG levels in mice immunized with vaccines adjuvanted with 5 or 7.5 μg GPI-0100 were similar to levels in sublethally infected mice. No IgA was found in the non-immunized but challenged control group, indicating again that the infection did not have substantial effects on antibody responses within the 5-day period post challenge.

### 3.3. Analysis of B Cell Responses

**Figure 3:**
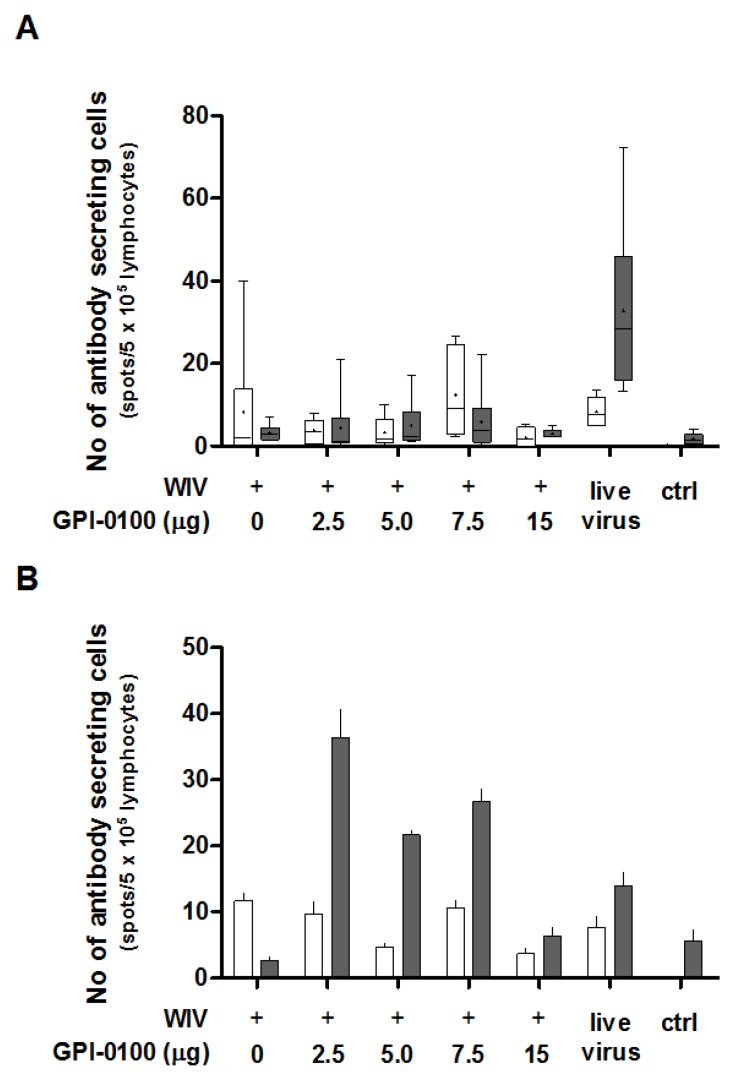
Effect of GPI-0100 on antigen-specific B cell responses. Effects of GPI-0100 dose on the numbers of A/PR/8 HA-specific B cells were evaluated in the mice described in the legend to Figure 1 five days after challenge. Splenocytes (**A**) and lung lymphocytes (**B**) were harvested and incubated overnight in plates coated with A/PR/8 subunit vaccine. The numbers of influenza-specific IgG (white)- and IgA (grey)-producing cells from (A) spleen and (B) lung were calculated by subtracting the number of spots formed in non-coated wells from the number of spots formed in coated wells. Live virus = sublethally infected, challenged mice. Ctrl = non-immunized, challenged mice. Splenocytes were analyzed for individual mice and data are depicted as box and whisker plots per experimental group. Lung lymphocytes were pooled per experimental group and data are depicted as mean + SEM of triplicate wells.

In order to determine the effect of GPI-0100 on the number of IgG- and IgA-producing B cells in the spleens and lungs found after challenge, antigen-specific B cell ELISpot assays were performed (Figure 3A,B). ELISpot results highlight that the number of spleen-resident B cells producing influenza-specific IgG was generally small and did not differ significantly between groups immunized with non-adjuvanted or GPI-0100-adjuvanted WIV, respectively (Figure 3A, white bars). No IgG-producing B cells were found in non-immunized but challenged control mice. This indicates that the antibody-secreting B cells present were the result of the prior vaccination rather than of the infection. Numbers of splenic B cells producing influenza-specific IgA were even lower and similar to those found in non-immunized, challenged control mice (Figure 3A, grey bars). Differences between mice immunized with adjuvanted and non-adjuvanted vaccine were therefore irrelevant. Only sublethally infected mice showed substantial numbers of IgA ASCs in the spleen.

In the lungs, the numbers of IgG ASC were again low for all groups (Figure 3B, white bars). However, IgA ASC were more abundantly present in all but the non-immunized control group and the group immunized with non-adjuvanted WIV (Figure 3B, grey bars). In all groups immunized with adjuvanted vaccine, the number of IgA-producing cells was higher than in the group immunized with non-adjuvanted vaccine. The numbers of IgA ASC in the lungs of mice vaccinated with 2.5, 5, 7.5 and 15 μg GPI-100 were approximately 13-, 8-, 10- and 2-fold higher than in the mice that received non-adjuvanted WIV. IgA ASC numbers in the groups that received GPI-0100-adjuvanted vaccine exceeded those found in lungs of sublethally infected mice (except for the 15 μg GPI-0100 group). Furthermore, the lungs of mice immunized with GPI-0100-adjuvanted formulations contained more antigen-specific IgA ASC than IgG ASC, while in the mice that received WIV alone, more IgG ASC than IgA ASC were detected.

**Figure 4:**
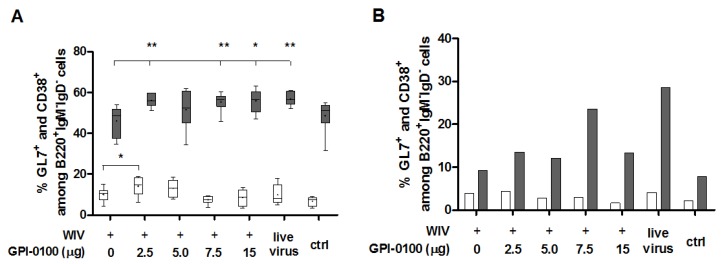
Effect of GPI-0100 on germinal center and memory B cell responses. Effects of GPI-0100 dose on germinal center (white) and memory (grey) B cells were evaluated in (**A**) splenocytes and (**B**) pooled lung cells of the mice described in the legend to Figure 1 five days after challenge. B cells in germinal centers were evaluated by determining the percentage of GL7^+^ cells among total class-switched B cells (B220^+^IgM^−^IgD^−^). Similarly, memory B cells were evaluated by determining the percentage of CD38^+^ cells among total class-switched B cells. Live virus = sublethally infected, challenged mice; ctrl = naïve, challenged mice. Splenocytes were analyzed for individual mice and data are depicted as box and whisker plots per experimental group. Lung lymphocytes were pooled per experimental group. Levels of significance are presented as * *p* < 0.05, ** *p* < 0.01.

Development of high-affinity memory B cells takes place in specialized lymphoid structures called germinal centers (GC). In the lungs, GC are found in bronchus-associated lymphoid tissue (BALT) [21]. To find out whether GPI-0100 had an effect on the number of B cells present in germinal centers, we enumerated in spleens and in lungs class-switched B cells carrying the germinal center marker GL7 [22,23] (Figure 4A,B). In general, the percentages of GL7^+^ cells among class-switched (IgM^−^IgD^−^B220^+^) B cells were low and there was no consistent difference in the number of these cells (neither in spleen nor in lungs) between mice immunized with WIV in absence or presence of GPI-0100, nor between immunized mice and convalescent or naïve mice (Figure 4A,B, white bars). In contrast, the percentages of CD38^+^ memory B cells (delineated as CD38^+^B220^+^IgM^−^IgD^−^ [24,25]) in spleens as well as in lungs were increased by GPI-0100, irrespective of the dose used (Figure 4A,B, grey bars). Percentages of memory B cells in mice immunized with adjuvanted vaccines were equal to those in infected mice. Enumeration of long-lived plasma cells (CD138^+^B220^+^IgM^−^IgD^−^) did not reveal any effect of immunization and infection on this cell type, neither in spleen nor in lung [26].

### 3.4. Analysis of Cytokine Responses

**Figure 5:**
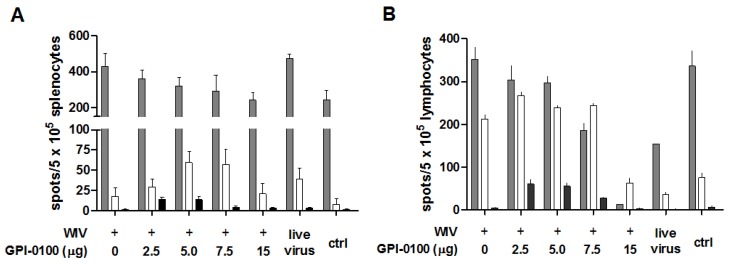
Effect of GPI-0100 on antigen-specific cellular immune responses. Effects of GPI-0100 dose on the number of A/PR/8-specific T cells were evaluated in the mice described in the legend to Figure 1 five days after challenge. Splenocytes (**A**) and lung lymphocytes (**B**) were incubated overnight with or without A/PR/8 WIV. The numbers of IFNγ- (grey), IL4- (white) and IL17- (black) producing influenza-specific cells in spleen and lung were calculated by subtracting the number of spots formed by non-stimulated cells from the number of spots formed by stimulated cells. Live virus = sublethally infected, challenged mice; ctrl = naïve, challenged mice. Results are expressed as spot-forming cells per 5 × 10^5^ cells. Splenocytes were analyzed for individual mice and data are depicted as mean + SEM. per experimental group. Lung lymphocytes were pooled per experimental group and data are depicted as mean +SEM. of triplicate wells.

We earlier observed that GPI-0100-adjuvanted vaccine administered via the pulmonary route induces IL4-secreting T cells but hardly any IFNγ-secreting T cells ([15] and unpublished observations). We now evaluated the effect of immunization on the cytokine response found after challenge as this might affect or reflect clinical symptoms. For this purpose, we immunized mice with non-adjuvanted and GPI-0100-adjuvanted WIV and analyzed the numbers of IFNγ-, IL4- and IL17-secreting cells in both spleen and lungs five days after challenge with live influenza virus (Figure 5).

High numbers of IFNγ-secreting cells were found in the spleens of all challenged animals (>200/5 × 10^5^ splenocytes). In mice immunized with GPI-0100-adjuvanted vaccines, the numbers of IFNγ-secreting cells were lower than in mice receiving WIV alone. This trend was GPI-0100 dose-dependent; yet, significance was only reached for the 15 μg adjuvant group. As expected, the spleens of sublethally infected, challenged mice contained high numbers of IFNγ-producing T cells. Substantial numbers were also found in the spleens of naïve mice 5 days after influenza virus challenge, indicating induction of IFNγ-producing cells by the infection.

Contrary to IFNγ-secreting cells, the number of IL4-secreting cells was increased in mice immunized with GPI-0100-adjuvanted WIV as compared to mice immunized with WIV alone. Mice in the 5 or 7.5 μg GPI-0100 groups had approximately three-fold more IL4-producing cells in the spleen than mice immunized with WIV alone (significant for 5 μg GPI-0100) while lower and higher amounts of the adjuvant had no effect. IL17-producing cells were present in spleens with low to very low frequency. Low doses of GPI-0100 (2.5 and 5 μg) increased the number of the IL17-producing cells significantly but due to the low overall numbers the relevance of this observation is unclear.

In the lungs, as in the spleens, the number of IFNγ-secreting cells decreased with increasing doses of GPI-0100; yet, the dose dependency was much stronger and no IFNγ-secreting cells were present in the lungs of mice from the 15 µg GPI-0100 group (Figure 5B). The naïve control group displayed substantial numbers of IFNγ-secreting cells, most probably representing T cells induced by the live virus infection given 5 days prior to sacrifice. The numbers of IL4-secreting cells in the lungs were generally high as compared to the numbers in the spleen. Immunized mice displayed much higher numbers of IL4-secreting cells in the lungs than previously infected and naïve, challenged mice. GPI-0100 did not affect these numbers except for the 15 μg GPI-0100 dose for which the level of IL4-secreting cells was low. Analysis of IL17-secreting cells from lungs showed that, compared to mice given WIV alone, mice immunized with 2.5, 5 or 7.5 µg GPI-0100 had approximately 12-, 11- and 5-fold more IL17-secreting cells. In mice immunized with 15 μg GPI-0100, the number of IL17-secreting cells was similar to that in the WIV alone group and the previously infected group.

Overall the analysis of cytokine-secreting cells in immunized and challenged mice indicates that GPI-0100 stimulates Th2 responses and suppresses Th1 responses in a dose-dependent manner. Induction of Th17 cells benefits from low, but not from high, doses of GPI-0100.

## 4. Discussion

The immune response to vaccination is determined by the antigen-adjuvant combination and the route of delivery. In previous studies, we showed that GPI-0100 is a potent adjuvant for intramuscular-delivered as well as for pulmonary-delivered subunit or WIV influenza vaccine [1,14,15]. In this study, we aimed to get more insight in the immune mechanisms stimulated by GPI-0100 adjuvantation of pulmonary-delivered dry powder vaccines and to determine the effect of the GPI-0100 dose on these immune mechanisms.

Pulmonary delivery of WIV with different amounts of GPI-0100 resulted in effective influenza-specific mucosal and systemic immune responses. GPI-0100 enhanced systemic IgG1 and mucosal IgA responses but had no effect on IgG2c responses. Compared to non-adjuvanted vaccine, GPI-0100-adjuvanted WIV gave rise to more antigen-specific IgA- but not IgG-producing B cells in the lungs along with better mucosal and systemic memory B cells responses. Intermediate doses of 5 μg or 7.5 μg GPI-0100 were most effective in enhancing humoral immunity. These doses of GPI-0100 were also optimal for stimulation of IL-4-producing T cells while the GPI-0100 dose was negatively correlated with the numbers of IFNγ-producing T cells.

We observed that the influenza-specific serum IgG titers increased with the amount of GPI-0100 present in the vaccine formulation. These results are in line with earlier observations of others and us, indicating that saponin-derived adjuvants delivered via the pulmonary route effectively stimulate antibody responses to admixed antigen in mice and in sheep [12,14,15]. Although after the first vaccination antibody titers elicited by GPI-0100-adjuvanted vaccines were low compared to titers induced by sub-lethal amounts of live virus, after two vaccinations, antibody titers equaled those in convalescent mice. These observations confirm that GPI-0100 exhibits strong adjuvant function in the lungs. Increased serum IgG responses in mice immunized with adjuvanted vaccines were not correlated with significant increases in splenic influenza-specific ASCs. Probably, ASCs were rather located in lymph nodes or in bone marrow four weeks after primary vaccination [27,28].

In line with previous observations, GPI-0100 did not change the IgG1-dominated antibody response to pulmonary-delivered WIV [15,16]. This indicates that GPI-0100 was unable to alter the anti-inflammatory Th2 microenvironment of the lungs, not even in C57Bl/6 mice, which are regarded as a prototypical Th1-type mouse strain [29,30]. In contrast, Ashtekar and co-workers did observe stimulation of IgG2a responses by GPI-0100 when immunizing mice by total respiratory tract administration with a protein-based *Francisella tularensis* vaccine [31]. However, these authors used a dose of 100 μg GPI-0100, which might explain their observations. Indeed it has been shown earlier that at low doses GPI-0100 stimulates mainly IgG1 responses, whereas at high doses (≥50 μg) both IgG1 and IgG2a responses are enhanced [7].

IgA is the predominant antibody at mucosal surfaces having the ability to neutralize free virus and intracellular virus in epithelial cells, thus providing protection against influenza infection of the respiratory tract [32]. Analysis of IgA in the nasal cavity highlighted that nasal IgA titers induced by GPI-0100-adjuvanted WIV were low compared to those induced by live virus infection. Active virus replication in the nasal cavity of infected mice leads to stimulation of antigen presenting cells (APC) in the nasal-associated lymphoid tissue (NALT). This results in programming of B cells such that memory B cells home to the nose, where upon infection they start to differentiate to IgA-producing plasma cells [25,33,34]. In contrast, during pulmonary immunization, the nasal cavity is bypassed by the vaccine which leads to stimulation of epithelial cells and APC in the lungs. In line with this, we found that GPI-0100 was capable of promoting amounts of lung IgA comparable to those induced by live virus infection. The high amounts of IgA correlated well with the presence of more influenza-specific IgA ASC and enhanced numbers of resident memory B cells in the lungs of mice immunized with adjuvanted vaccine. However, B cells primed in the lung might not have the capacity to home to the nose upon infection, which would explain the low levels of nasal IgA in the immunized animals. We observed that GPI-0100 enhanced the levels of lung IgG to levels elicited by live virus. However, no differences were found in the number of lung ASC-producing influenza-specific IgG between mice immunized with plain and with GPI-0100-adjuvanted vaccine. Presumably, most IgG reaches the lungs by transudation from serum rather than being locally produced [35]. The relative contribution of IgA and IgG in the lung to protection against influenza virus is not entirely clear. On the one hand, IgA ASC were shown to proliferate rapidly upon reinfection [25]. On the other hand, IgG in the lung was shown to be more important than IgA for protection against influenza [36].

Germinal centers (GC) are important for class-switching of B cells, somatic hypermutation, generation of long-lived plasma cells and differentiation of memory B cells [37]. The number of splenic GC has earlier been shown to correlate with memory B cell formation and longevity of antibody responses upon immunization with adjuvanted antigen [38]. Moreover, pulmonary GC induced by influenza infection were shown to contribute decisively to local immune responses and protection [39]. Pulmonary immunization with plain or GPI-0100-adjuvanted WIV induced only slight increases in the number of GL7^+^ B cells in spleens and had no effect on the number of these cells in the lungs. Since we analyzed lung and spleen cells five days after challenge with influenza virus, GC might have been induced by the infection and these GC might have obscured earlier existing vaccine-induced GC. Yet, the low number of GL7^+^ cells found in spleens and lungs does not support this hypothesis. While GPI-0100 adjuvantation had little effect on GC B cells, it significantly increased the number of CD38^+^ memory B cells in both spleens and lungs. Even the lowest adjuvant dose was effective in this respect. Memory B cells, especially memory B cells in the lungs, have been associated with improved protection from influenza infection [25].

Adjuvants have the ability to qualitatively alter the immune response to an accompanying antigen by driving APC to release sets of cytokines which promote polarization of naïve T cells to distinct T-helper phenotypes [40]. IFNγ, IL4 or IL17 are key cytokines secreted by Th1, Th2 or Th17 cells, respectively. Th1 and Th2 cells stimulate B cells to produce IgG2a/c and IgG1 antibodies, respectively [37]. Th17 cells are implicated in recruitment of neutrophils to the lungs [41]. Recently, it was shown that Th17 cells, which enter the germinal center cycle, can convert into IgA-inducing T follicular helper (Tfh) cells [42]. We found a clear negative correlation between the GPI-0100 dose in the vaccine and the number of IFNγ-producing T cells present in spleen and lungs. Yet, since T cells were measured in immunized and challenged mice, it is unclear whether lower numbers of IFNγ-producing T cells were due to poor induction by vaccination or, rather, reflected inhibition of virus replication in the lungs. On the other hand, we found increased numbers of IL4-producing cells in the spleen with increasing GPI-0100 doses, while in the lungs GPI-0100 had no effect on IL4-producing T cells. Together with stimulation of IgG1 rather than IgG2c responses, these results suggest that at the doses used GPI-0100 delivered via the pulmonary route promotes mainly Th2 responses. Previous studies suggest that the microenvironment in the respiratory tract favors a Th2 immune response resulting in release of IL2, IL4, IL5, IL10 and TGFβ [29,43]. Th17 responses were increased at low, but not at higher, doses of GPI-0100 and effects were more pronounced in the lungs than in the spleens. However, compared to IFNγ- and IL4-producing T cells, the numbers for IL17-producing T cells remained low. Induction of IL17 cells has been shown to determine the fate of pathogens upon pulmonary infection via neutrophil recruitment and promotion of IgA production [41,42]. The role of Th17 cells in influenza is still debated. Several studies report Th17 cells or the presence of IL17 to be beneficial [44,45]. Yet, other studies report a detrimental effect of vaccine-induced Th17 [46,47].

One aim of our study was to get insight into dose-dependent effects of GPI-0100. Interestingly, most immune responses were found to be highest at intermediate doses of 5 and 7.5 μg GPI-0100. In general, the doses chosen for our study were rather low compared to the amounts used by others. We argued that in order to minimize the risk of detrimental effects of the saponin adjuvant on lung tissue, the dose should be as low as possible. In earlier studies, we showed that a dose of 15 μg GPI-0100 did not change the histological appearance of the lung and had no effect on the number of infiltrating neutrophils and macrophages [14]. Nevertheless, we cannot exclude completely that the low levels of antibody- and cytokine-secreting cells found in the 15 μg GPI-0100 group in the current study are due to toxicity induced by the adjuvant. C57BL/6 mice might be more susceptible to interventions in the lungs and thus to detrimental effects of the adjuvant than Balb/c mice used in our previous studies. Indeed, C57BL/6 were shown to exhibit more pronounced recruitment of eosinophils and neutrophils upon OVA exposure as compared to Balb/c mice [48]. Also, genetic heterogeneity leads to differences in cytokine/chemokine release between C57BL/6 and Balb/C mice upon antigen exposure which ultimately affect immune responses [30]. Despite the low amounts of GPI-0100 used, the adjuvant had prominent effects on the induced immune responses, in particular on antibody and B cell responses.

## 5. Conclusions

Our results show that GPI-0100, when administered together with antigen via the pulmonary route, is an excellent adjuvant for modulating mucosal and systemic immune responses. GPI-0100 is capable of stimulating IgA responses in the respiratory tract by promoting resident IgA ACS in the lungs. In addition, GPI-0100 enhances mucosal and systemic IgG responses. This is reflected by increased numbers of memory B cells in the lung and spleen that can proliferate and differentiate rapidly to antibody-secreting plasma cells upon pathogen encounter. Thus, GPI-0100 is a promising adjuvant for use in pulmonary vaccines.
